# A Case for Help

**Published:** 1888-11-10

**Authors:** A. E. Young

**Affiliations:** Matron. Ironmongers' Almshouses, Kingsland Road


### A CASE FOR HELP.

Sir,—May I appeal to the benevolent and charitably disposed readers of your valuable and influential paper, for assistance in the following case, the deserving nature of which I can thoroughly guarantee: Chas. Bridgett (age 39, married, two children), has for two years been unable to work, owing to consumption and bronchitis; he had a post on the railway, to which, if his health improved, he might return. The doctors advise that a stay of three months at a seaside convalescent hospital would be likely to be of much service. To effect this, a sum of about £7 is required, as the hospital to which it is proposed C. B. should go requires an entrance fee and a weekly payment of 10s. I have a governor's letter, medical certificate, etc., for this patient, but we are at a standstill for the above sum. I should be pleased to furnish further particulars, or references, if desired. A. E. Young, Matron.
Ironmongers' Almshouses, Kingsland Road,
November 6th, 1888.

